# Development and validation of a next-generation sequencing-based method for calculating the breast cancer polygenic risk score PRS_313_

**DOI:** 10.1016/j.breast.2025.104580

**Published:** 2025-09-16

**Authors:** Flora Ponelle-Chachuat, Mathis Lepage, Sandrine Viala, Mikaïl Kelleci, Edith Le Floch, Claire Dandine-Roulland, Delphine Bacq, Robert Olaso, Jean-François Deleuze, Nancy Uhrhammer, Mathilde Gay-Bellile, Yannick Bidet, Maud Privat

**Affiliations:** a Université Clermont Auvergne, INSERM, U1240 Imagerie Moléculaire et Stratégies Théranostiques, Clermont Ferrand, France; b Département d’Oncogénétique, Centre Jean Perrin, Clermont-Ferrand, France; c Centre National de Recherche en Génomique Humaine, Institut de Biologie François Jacob, CEA, Université Paris-Saclay, Evry-Courcouronnes, France

**Keywords:** Polygenic risk score, Breast cancer, NGS, OncoArray, PRS_313_

## Abstract

Polygenic risk scores (PRS) are genetic tools that quantify an individual's predisposition to certain diseases by combining the effects of many genetic variants. The PRS_313_ includes 313 genomic variants and has been incorporated into the BOADICEA prediction model and in the CanRisk software to refine breast cancer risk. However, its current implementation relies on SNP microarrays, limiting its use in sequencing-based clinical workflows.

In this study, we directly compared SNP microarray technology and targeted NGS sequencing to determine the PRS_313_. The two methods were tested for 154 patients. To replace PRS_313_ SNPs with low sequencing coverage and/or in regions of low complexity, 27 proxy SNPs in high linkage disequilibrium were integrated into the panel. After this optimization, the NGS-derived PRS_313_ demonstrated strong concordance with the microarray reference (R^2^ = 0.95), with sensitivity and specificity reaching 96 % and 97 %, respectively. Moreover, the clinical risk category, as defined by CanRisk, remained consistent in 97 % of cases across both methods.

These findings validate the use of targeted NGS for PRS_313_ calculation, demonstrating its feasibility, accuracy, and potential for easy integration into routine oncogenetic workflows. By enabling PRS calculation from the same sequencing data used for gene panel testing, this approach eliminates the need for separate genotyping platforms, offering a cost-effective and clinically practical solution to support the broader implementation of personalized breast cancer risk prediction.

## Highlights

• We developed a targeted NGS method to calculate breast cancer PRS_313_.
• A 96 % of sensitivity and 97 % of specificity is achieved post-optimization.
• PRS calculated by our optimized NGS panel matches Oncoarray PRS (R^2^ = 0.95).
• Risk categories of breast cancer remain consistent in 97 % of cases across both methods.
• Our method enables PRS calculation during routine oncogenetic testing.

## 1 Introduction

Polygenic risk scores (PRS) are a powerful tool in genetics to quantify an individual's genetic predisposition to certain traits or diseases based on the cumulative effect of many genetic variants. Each variant contributes a small amount to the overall risk, and together, these scores can help predict the likelihood of developing conditions such as heart disease, diabetes, and various forms of cancer. Large-scale genome-wide association studies have thus identified hundreds of single nucleotide polymorphisms (SNPs) linked to breast cancer. Several PRS have been developed specifically for breast cancer, each based on different sets of genetic variants and aimed at improving risk assessment and management.

Mavaddat et al. defined a PRS that includes 313 genomic variants (267 SNPs and 46 indels) and is more predictive than the PRS based on 77 SNPs previously reported. The lifetime risk of breast cancer for women in the highest percentile for this PRS_313_ was 32 % [1]. Validation of PRS_313_ was carried out on several independent prospective cohorts [[1], [2], [3], [4]]. In particular, Jiao et al. showed that PRS_313_ may be of clinical utility for women with a strong family history of breast cancer without pathogenic BRCA variant [5]. PRS_313_ was therefore introduced into the BOADICEA breast cancer risk prediction model [6]. This algorithm, available via the CanRisk tool (https://CanRisk.org) [7,8], integrates clinical data (age, sex, hormonal exposure, breast density, etc.), familial data (personal or family history of HBOC spectrum cancers) and genetic data (mutations in predisposition genes and PRS). The CanRisk tool has been widely validated in prospective studies and it was shown that among the individual model components, the PRS_313_ contributed most to breast cancer risk stratification in the general population [2,3].

At present, PRS are not yet used in routine clinical practice, but large cohorts are currently being studied to determine whether polygenic risk factors can be taken into account. WISDOM in the United States, MyPeBS in Europe, PRiMO in Australia [9] and PRISMA in Spain [10] are examples of clinical studies that evaluate personalized screening programs based on each woman's individual risk of developing breast cancer. The aim of these clinical studies is to evaluate whether personalized breast cancer screening could be a better screening strategy than the age-based protocol currently recommended.

In clinical practice, patients with family history of breast cancer are referred to an oncogenetics consultation, where they are screened for mutations in predisposition genes. Technically, this mutation search is carried out by sequencing, and if PRS becomes a clinical routine, it would be interesting to be able to calculate them using the same technique. This would save oncogenetics laboratories time and money, and avoid major investments in SNP array equipment. For the moment, however, PRS have been only validated by SNP microarray detection, which is the fastest and cheapest technique for the large cohorts required for PRS validation. Some studies analyzed the possibility to introduce PRS in NGS panel sequencing [11,12] or exome sequencing [13]. Nevertheless, we could not find data about direct comparison of PRS calculated by NGS and by microarrays including imputation.

The aim of our study is therefore to build an accurate method of calculating the PRS_313_ by targeted NGS sequencing, and to compare our scores to those calculated by microarray. This would validate this methodological approach and enable it to be used in most oncogenetic laboratories.

## 2 Material and methods

• Sample selection

All women were enrolled by the oncogenetic consultation of the Centre Jean Perrin, for molecular diagnostic of hereditary predisposition of breast cancer, between March 2023 and June 2024. Because our study focuses on the genetic concordance between our NGS design and the original array technique, we included the patients regardless of their age, genetic and cancer status or ethnic origin. 154 DNAs, selected for their wide range of PRS scores determined by NGS, were sent to the Centre National de Recherche en Génomique Humaine (CNRGH) for genotyping on Infinium OncoArray Illumina microarrays.

Patients signed informed consent, and study ethics approval was obtained on June 24, 2024 (CJP IRB committee, IRB 00013468).

• PRS determination by SNP microarray

Genotyping of the cohort was performed at CNRGH. Before genotyping, quantification was systematically performed on the samples (Quant-It kits, Thermofischer). An assessment of the quality of the DNAs was performed for 10 % of the samples by PCR amplification and DNA migration on Tapestation 4200 (Agilent).

After quality control, DNAs were aliquoted in 96-well plates (JANUS liquid handling robot, PerkinElmer) for genotyping; sample tracking was ensured by barcode scanning for each sample. Two DNA positive controls were systematically inserted in a random fashion into the plates. Genotyping was performed on a high throughput Illumina automated platform, using the Infinium OncoArray. Standard automated protocols from Illumina ® were performed (Illumina ®, San Diego, USA). Reading of the chips was performed on iScan + scanners (Illumina®, San Diego, USA) and primary analysis of the results was done using GenomeStudio software (Illumina®, San Diego, USA).

The analysis of the internal controls provided by Illumina and the randomly distributed positive controls allowed the validation of the technological process. The quality control of the genotypes was performed for each marker by measuring the deviation from the expected Hardy-Weinberg genotypic proportions for each individual, by measuring the success rate, the average heterozygosity and detecting duplications and outliers.

Similarly to the process described in Mavaddat et al., imputation was performed from the OncoArray using the Phase 3 release v5 of the 1000 Genomes data as reference. We followed a two-stage approach using SHAPEIT2 [14] for phasing and Minimac4 [15] for imputation.

For each variant, the most probable genotype was converted into 0, 1 or 2 sought allele, and this value was multiplied by its beta coefficient. The polygenic risk score was calculated as the sum of all 313 products.

• PRS determination by targeted sequencing

DNA was extracted from blood using automated QIAsymphony extraction (Qiagen, Hilden, Germany). DNA was fragmented enzymatically as part of the Kapa library kit. Kapa HyperPlus library preparation and HyperExplore probes and reagents (Roche, Bâle, Switzerland) were used for library preparation and capture. Quality of fragmentation, library and capture were controlled using a Tapestation 4200 instrument (Agilent, Santa Clara, CA, USA). Sequencing was performed using 300-cycle Illumina kits with NextSeq 550 or 2000 Instruments (Illumina, San Diego, CA, USA). All steps were performed following providers’ guidelines, aiming for a minimal depth of 30X on each target. To increase general specificity, probes covering 20bp on each side of all 313 SNPs were spiked into the panel routinely used for molecular diagnostics of hereditary predisposition to cancer in the Centre Jean Perrin and the data were generated to meet the medical laboratory requirements [16]. Only 313-SNP regions were considered for this study. The PRS was calculated exactly as for the microarray, using the genotype determined by GATK.

• Alternative SNP design for NGS

Alternative SNPs were chosen with the LDproxy Tool of the NIH [17], using the CEU population and a 50 kb window. Three SNPs in linkage disequilibrium with each uncovered SNP of the original PRS_313_ were selected according to the highest disequilibrium (D′) and correlation (R^2^) values. Genomic context was observed in UCSC Genome Browser to exclude repeats and low complexity regions and the best two were added to the revised design. D’ and R^2^ values of the alternative SNP selected in our optimized design are mentioned in the Supplementary Table 1.

• Bioinformatic analysis

Alignment was performed on the Genome Reference Consortium Human Genome Build 37 (GRCh37 hg19) using Burrows-Wheeler Aligner (v0.7.17). Genome Analysis Toolkit 4.2.6 (GATK) and PICARD 2.26 tools were used for base quality score recalibration, realignment and PCR duplicates removal. Variant calling was performed using GATK HaplotypeCaller.

## 3 Results

For 154 patients, PRS_313_ was determined both by targeted sequencing and by SNP microarray.

• Efficiency of SNP sequencing

The original version of our PRS panel reached a global mean depth of 559x. For 289 SNPs, the coverage was sufficient for all the patients (mean depth>100x) but 15 SNPs had a null or very low depth (mean < 30x, Fig. 1 and Supplementary Table 1). Most SNPs with low sequencing depth were found in regions of the genome with low complexity, such as homopolymers or repeated regions. These regions are known to reduce the accuracy of NGS. Moreover, 46 SNPs showed concordance with OncoArray results below 90 %, including the 15 low-covered SNPs.

**Fig. 1:**
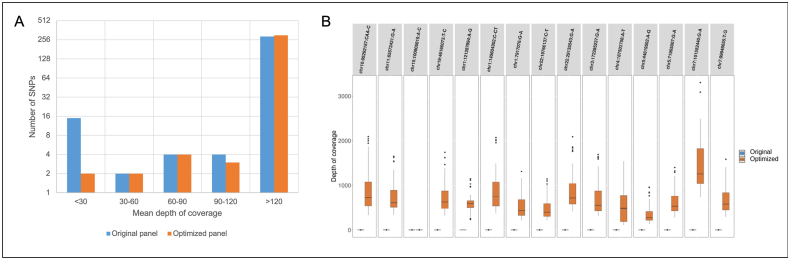
Average sequencing depth of the 313 SNPs PRS_313_ was determined by targeted sequencing on an original panel and on an optimized panel. A- The average depth of coverage was calculated for each 313 SNPs on the original panel (blue) and the optimized panel (orange). The histogram shows the distribution of the 313 SNPs according to their reading depth. B- The 15 SNPs with an average depth <30x on the original panel are detailed in boxplots, representing the distribution of read depth according to patients, for the original panel (blue) and the optimized panel (orange). (For interpretation of the references to colour in this figure legend, the reader is referred to the Web version of this article.)

For these 46 genomic variants with low-depth and/or low-concordance, a second version of the PRS panel was designed by adding SNPs in linkage disequilibrium, using LDproxy Tool of the NIH. For each original SNP, concordance and sequencing depth of all new SNPs were compared, and only the best was selected, if better than the original. Our optimized PRS panel finally included 27 modified SNPs (r^2^ between 0.47 and 1; D’ between 0.7998 and 1). Two of these SNPs were in negative linkage disequilibrium, the genotypes detected in NGS must therefore be reversed (Supplementary Table 1). This optimized PRS panel decreased the number of low depth SNPs to 2 with a mean < 30x (position and weight in the PRS in Supplementary Table 1). Indeed, none of the selected SNPs in linkage disequilibrium with chr15:100905819:A > C could be sequenced. Another SNP (chr1:121287994:A > G) in linkage disequilibrium showed good sequencing depth but correlated poorly with the genotype given by the array and was therefore not retained in our optimized panel. For these 2 SNPs without sequencing data, the wild-type allele was used as it is the most frequent in the general population.

• Concordance between array and NGS genotypes

**Fig. 2:**
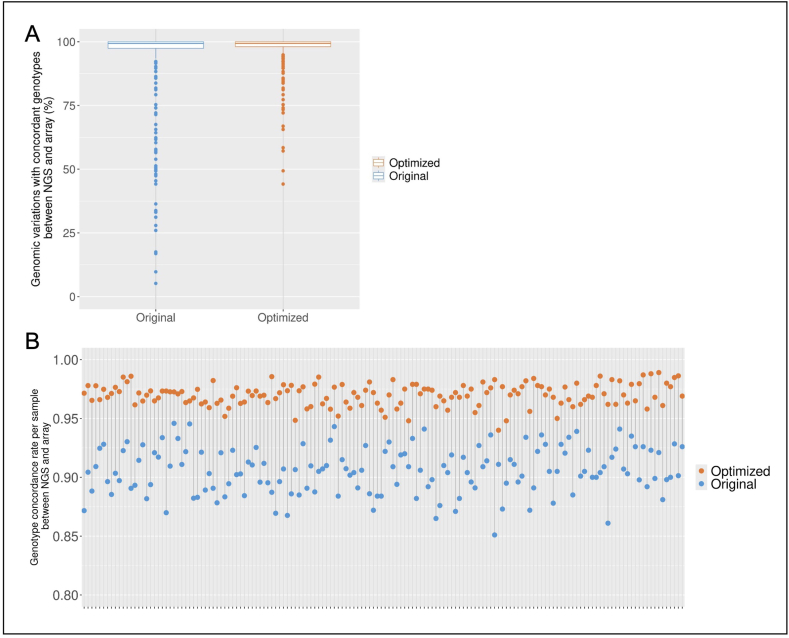
Genotypic concordance of the 313 SNPs of the PRS determined by OncoArray or NGS PRS_313_ was determined both by microarray genotyping and by targeted sequencing on the original panel and on the optimized panel. For the original panel (blue) and the optimized panel (orange), the genotype concordance between OncoArray and NGS genotyping was calculated for each SNP (A) and for each patient (B). (For interpretation of the references to colour in this figure legend, the reader is referred to the Web version of this article.)

We then evaluated if the genotypes determined by NGS and microarrays were concordant (Fig. 2).

We used Illumina OncoArray that included 105 genotyped SNPs and allowed imputation of the remaining 208 SNPs, as published in Mavaddat et al. [1] (Supplementary Table 1).

For each of the 313 SNPs, we looked for the percentage of patients for whom the genotype (non-carrier, heterozygous or homozygous) matched between genotyping and sequencing (Fig. 2A). For the original PRS (blue), 151 SNPs (48 %) were concordant (same genotype) between the Oncoarray and NGS for all patients and 267 SNPs (85 %) were concordant for over 90 % of patients. However, 18 SNPs were discordant for more than 50 % of patients, including 7 SNPs with poor coverage. The optimized version of our PRS panel (orange) increased genotype concordance, with 287 SNPs (92 %) concordant for more than 90 % of patients and only two of the 313 SNPs were discordant for more than 50 % of patients. All variants showing concordance below 90 % were imputed on the OncoArray. Especially, five SNPs (chr8:128213561:C-CA, chr5:58241712:C-T, chr1:217053815:T-G, chr8:143669254:A-G and chr15:66630569:G-A) showed poor genotype concordance rates between NGS and microarray despite a high quality of sequence by NGS (mean depth ≥100X, quality score ≥30 and non-repetitive region). The discrepancy is most likely due to incorrect imputation.

For each patient, we then looked at the percentage of SNPs that were concordant between array and NGS (Fig. 2B). The percentage of concordant SNPs was very homogeneous between the different samples, excluding DNA quality problems. The optimized version of our PRS panel improved the results, rising from 85 to 95 % of matching SNPs to 94–99 % of matching SNPs.

In addition, we calculated the sensitivity and specificity of the NGS method for determining the 313 genotypes on all 154 patients, considering OncoArray as the reference method (Table 1). With the original panel, we obtained a sensitivity of 88 % and a specificity of 97 % and the optimized panel increased sensitivity to 96 % without decreasing specificity. For the SNPs directly on the array (to exclude imputation bias), the sensitivity and specificity both reached 99.8 %.

• Allelic frequencies of the SNPs

**Table 1:** Sensitivity and specificity of the NGS method of PRS determination.

	All SNP		Non imputed SNP	
	NGS Original	NGS optimized	NGS Original	NGS optimized
True positive	22117	24030	8687	8687
True negative	22553	22654	7456	7456
False positive	679	662	12	12
False negative	2853	856	15	15
Sensitivity (%)	88.57	96.56	99.83	99.83
Specificity (%)	97.08	97.16	99.84	99.84
Positive predictive value (%)	97.02	97.32	99.86	99.86
Negative predictive value (%)	88.77	96.36	99.80	99.80

TP: true positive; TN: true negative; FP: false positive; FN: false negative.

Sensitivity corresponds to (TP∗100)/(TP + FN).

Specificity corresponds to (TN∗100)/(TN + FP).

Positive predictive value corresponds to (TP∗100)/(TP + FP).

Negative predictive value corresponds to (TN∗100)/(TN + FN).

**Fig. 3:**
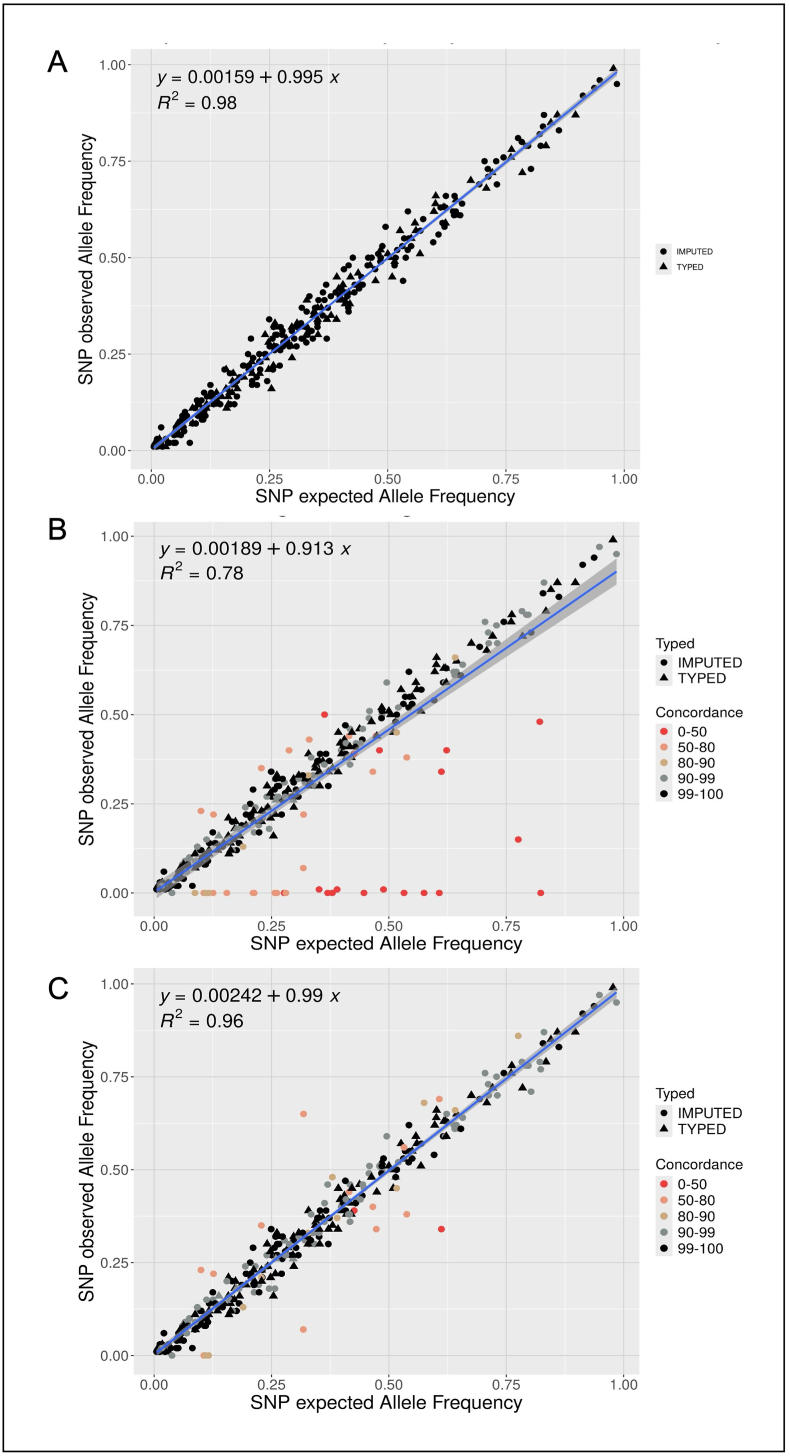
Comparison of observed allele frequencies with published frequencies Comparison of the frequency of the 313 SNPs found in the 45,494 female controls of Mavaddat et al. [1] with the allelic frequencies in our array data (A), in the original panel (B) and in the optimized panel (C). The regression lines were plotted in blue and the 95 % confidence interval in grey. (For interpretation of the references to colour in this figure legend, the reader is referred to the Web version of this article.)

To rule out any bias in our population, we looked at the allelic frequency obtained by NGS or microarray of each SNP of the PRS in our 154 patients. These allelic frequencies were compared with those found in the 45,494 female controls of European ancestry genotyped with the OncoArray published in Mavaddat et al. [1]. For SNPs detected by array, allele frequencies correlated very well with expected frequencies (Fig. 3A, R^2^ = 0.98). The frequencies observed with our original PRS panel showed a drop in the correlation, because of the incorrectly sequenced SNPs located in low-complexity regions (Fig. 3B, R^2^ = 0.78). The optimized PRS panel increased this correlation close to that of the array (Fig. 3C, R^2^ = 0.96).

• Correlation between array-genotyped PRS and NGS PRS, and effect on cancer risk

**Fig. 4:**
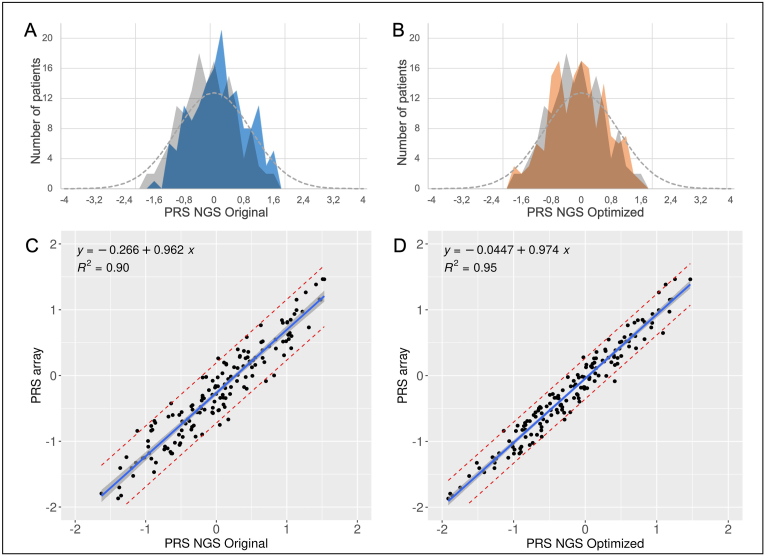
Comparison of PRS_313_ obtained between microarrays and NGS PRS_313_ was determined both by microarray genotyping and by targeted sequencing. The distribution of patients according to their PRS was represented for the original panel (in blue on panel A), for the optimized panel (in orange on panel B), and for the OncoArray (in grey on panels A and B). The dotted curve (panels A and B) represents a normal distribution of PRS between the extreme theoretical values of −4 and +4. The PRS obtained with the original panel (C) or the optimized panel (D) were compared to the PRS determined by microarray genotyping. The regression lines were plotted in blue and the 95 % confidence interval in grey. (For interpretation of the references to colour in this figure legend, the reader is referred to the Web version of this article.)

Polygenic risk scores were then determined using the 313 SNPs genotyped either by targeted sequencing or by OncoArray. The OncoArray PRS313 of our 154 patients ranged from −1.869 to 1.4655, meaning that we covered the PRS values of 90 % of the general population (Fig. 4A and B). The coefficient of determination R^2^ obtained between the microarray PRS and the NGS PRS is 0.90 on the original panel (Figs. 4C) and 0.95 on the optimized panel (Fig. 4D). There does not appear to be any further difference for extreme PRS.

**Fig. 5:**
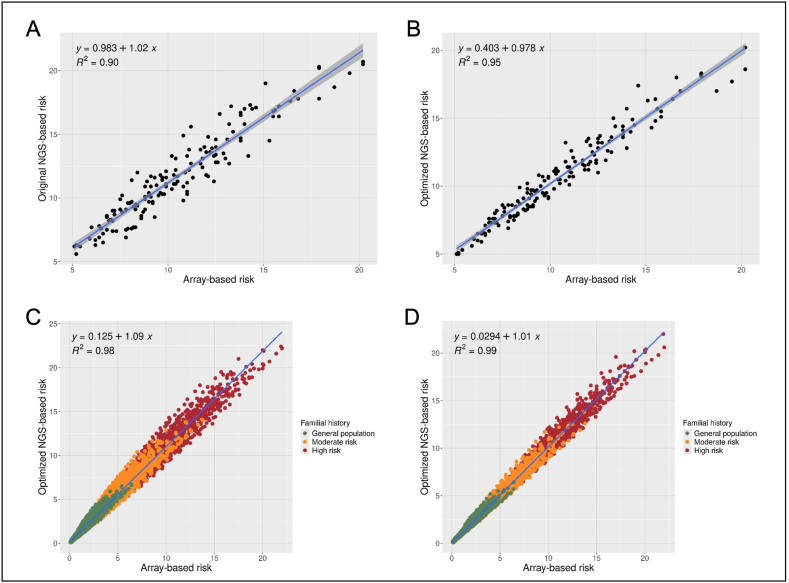
Concordance between cancer risks calculated with CanRisk, using the OncoArray PRS or the NGS PRS Cancer risks were calculated with CanRisk, using OncoArray-genotyping or sequencing PRS. For 154 patients, a lifetime risk of breast cancer was calculated without any clinical data to maximise the effect of PRS. All were assumed to be 30 years old, with no familial history. For each patient, this risk was compared between the microarray PRS and the original panel PRS (A) or the optimized panel PRS (B). Then, 48 fictitious clinical situations cancer-free women were created by varying family history, height, weight, hormonal context, number of children, and breast density. In particular, a family history representative of the general population (no cases of breast cancer), of a moderate risk (two cases of breast cancer in a sister (45) and mother (56)), and high risk (four cases of breast cancer in two sisters (32 and 35), mother (45), and grandmother (50)) were simulated. The 10-year risk of breast cancer was then assessed in CanRisk in these 48 clinical situations for the 154 PRS calculated by both OncoArray and NGS. The resulting risks were compared between the OncoArray PRS and the original panel PRS (C) or the optimized panel PRS (D). The regression lines were plotted in blue and the 95 % confidence interval in grey. (For interpretation of the references to colour in this figure legend, the reader is referred to the Web version of this article.)

Finally, we tested how the PRS, determined either by NGS or OncoArray, modified the risk of breast cancer calculated by CanRisk. Unfortunately, we were unable to calculate the actual risk of primary breast cancer for our patients using CanRisk because most of them had already developed breast cancer. Thus, to focus on the effect of their PRS, we first calculated the risks for all 154 patients, without considering age, clinical, familial and monogenic risk factors. We therefore entered only the PRS in the CanRisk software, with a fictitious and arbitrary age of 30 for all patients. The determination coefficient R^2^ was 0.90 for the original panel and 0.95 for the optimized panel, considering the lifetime breast cancer risk (Fig. 5A & B). For this cohort, we determined for each patient the lifetime risk category as defined by the British National Institute for Health and Care Excellence [18] (Table 2). With the original panel, 10 patients (6 %) were not classified in the same risk category by the two PRS calculation methods. Our optimized panel lowered these differences to 3 patients (2 %). These three patients presented a risk of the general population according to the PRS Oncoarray, but a moderate risk of breast cancer according to our PRS NGS, however with values very close to the threshold (14.6 vs 17.4, 16.6 vs 18.0 and 16.8 vs 17.0 %).

**Table 2:** Lifetime breast cancer risk categories of the 154 patients when considering only PRS calculated either by OncoArray or by NGS (original or optimized panel).

	Oncoarray PRS	NGS original PRS	NGS optimized PRS
General population risk (<17 %)	147	137	144
Moderate risk (≥17 % and <30 %)	7	17	10
High risk (≥30 %)	0	0	0
Nb changes in risk category (%)	Ref	10 (6 %)	3 (2 %)
Total nb patients	154	154	154

The lifetime risk of breast cancer was calculated using the CanRisk tool (https://www.canrisk.org/), considering women aged 30 and incorporating PRS as the only risk factor. No other clinical or epidemiological data were entered.

According to the British National Institute for Health and Care Excellence, lifetime breast cancer risk was considered near-population when it was less than 17 %, moderate when it was between 17 % and 30 %, and high when it was greater than 30 % [18].

We then tested whether our PRS by NGS modified breast cancer risk by adding fictitious clinical and epidemiological data to approximate real-life conditions. We simulated 48 different clinical scenarios by varying family history, BMI, hormonal context, and breast density (Table 3). We applied these scenarios to 40-year-old women who had no identified pathogenic variants in predisposition genes. CanRisk was batch-loaded with all these data and the 154 PRS calculated in our patients either by OncoArray, original or optimized NGS panel. The 10-year breast cancer risk was calculated for all these 7392 fictitious clinical situations (154 PRS scores multiplied by 48 clinical situations). Once again, the correlation with OncoArray was better for the optimized panel (Fig. 5D, R^2^ = 0.99) than for the original panel (Fig. 5C, R^2^ = 0.98). We then investigated if the PRS calculated by NGS modified the 10-year breast cancer risk category, as defined by the British National Institute for Health and Care Excellence [18] (Table 3). Among the 7392 fictitious clinical situations, adding the reference PRS score (Oncoarray) to CanRisk changed the risk category for 1477 cases (20 %). Compared to the Oncoarray PRS, inclusion of the NGS PRS resulted in a change in risk category for 540 cases (7.3 %) for the original panel and 234 cases (3.2 %) for the optimized panel. For 96.8 % of the clinical situations, our NGS PRS would thus led to the same clinical follow up as the PRS calculated with OncoArray.

**Table 3:** 10-year breast cancer risk categories of the 154 patients when considering PRS calculated either by OncoArray or by NGS in 48 representative fictitious clinical situations.

						Change of risk category when adding Oncoarray PRS		Change of risk category with original panel PRS (compared to Oncoarray PRS)		Change of risk category with optimized panel PRS (compared to Oncoarray PRS)	
Family history	BMI	Contraceptive pill	Children number	Breast density	10-year risk without PRS (%)	Number of patients/154	%	Number of patients/154	%	Number of patients/154	%
General population No cases of breast cancer	23	No	0	A	0.5	0	0 %	0	0 %	0	0 %
	23	No	0	D	2.7	20	13 %	15	10 %	4	3 %
	23	No	3	A	0.4	0	0 %	0	0 %	0	0 %
	23	No	3	D	2.2	10	6 %	14	9 %	3	2 %
	23	Yes	0	A	0.7	0	0 %	0	0 %	0	0 %
	23	Yes	0	D	3.5	104	68 %	21	14 %	9	6 %
	23	Yes	3	A	0.6	0	0 %	0	0 %	0	0 %
	23	Yes	3	D	2.9	27	18 %	17	11 %	5	3 %
	30	No	0	A	0.5	0	0 %	0	0 %	0	0 %
	30	No	0	D	2.4	15	10 %	12	8 %	5	3 %
	30	No	3	A	0.4	0	0 %	0	0 %	0	0 %
	30	No	3	D	2	7	5 %	4	3 %	3	2 %
	30	Yes	0	A	0.6	0	0 %	0	0 %	0	0 %
	30	Yes	0	D	3.1	34	22 %	21	14 %	12	8 %
	30	Yes	3	A	0.5	0	0 %	0	0 %	0	0 %
	30	Yes	3	D	2.6	18	12 %	17	11 %	5	3 %
Moderate risk 2 cases of breast cancer in sister (45) and mother (56)	23	No	0	A	1.6	0	0 %	0	0 %	0	0 %
	23	No	0	D	8	33	21 %	21	14 %	10	6 %
	23	No	3	A	1.3	0	0 %	0	0 %	0	0 %
	23	No	3	D	6.7	40	26 %	23	15 %	15	10 %
	23	Yes	0	A	2.2	0	0 %	0	0 %	0	0 %
	23	Yes	0	D	10.3	112	73 %	21	14 %	11	7 %
	23	Yes	3	A	1.8	0	0 %	0	0 %	0	0 %
	23	Yes	3	D	8.7	28	18 %	23	15 %	8	5 %
	30	No	0	A	1.5	0	0 %	0	0 %	0	0 %
	30	No	0	D	7.3	33	21 %	23	15 %	12	8 %
	30	No	3	A	1.2	0	0 %	0	0 %	0	0 %
	30	No	3	D	6.1	51	33 %	21	14 %	11	7 %
	30	Yes	0	A	1.9	0	0 %	0	0 %	0	0 %
	30	Yes	0	D	9.4	127	82 %	23	15 %	9	6 %
	30	Yes	3	A	1.6	0	0 %	0	0 %	0	0 %
	30	Yes	3	D	7.9	32	21 %	26	17 %	12	8 %
High risk 4 cases of breast cancer in 2 sisters (32 and 35), mother (45) and grand-mother (50)	23	No	0	A	2.9	10	6 %	14	9 %	3	2 %
	23	No	0	D	14.2	44	29 %	19	12 %	8	5 %
	23	No	3	A	2.4	0	0 %	0	0 %	0	0 %
	23	No	3	D	11.9	78	51 %	25	16 %	8	5 %
	23	Yes	0	A	3.9	105	68 %	22	14 %	11	7 %
	23	Yes	0	D	18.1	10	6 %	6	4 %	3	2 %
	23	Yes	3	A	3.2	137	89 %	14	9 %	5	3 %
	23	Yes	3	D	15.3	30	19 %	20	13 %	10	6 %
	30	No	0	A	2.6	4	3 %	3	2 %	2	1 %
	30	No	0	D	12.9	65	42 %	26	17 %	12	8 %
	30	No	3	A	2.1	0	0 %	0	0 %	0	0 %
	30	No	3	D	10.8	99	64 %	26	17 %	9	6 %
	30	Yes	0	A	3.5	126	82 %	18	12 %	6	4 %
	30	Yes	0	D	16.5	23	15 %	15	10 %	10	6 %
	30	Yes	3	A	2.8	8	5 %	11	7 %	2	1 %
	30	Yes	3	D	14	47	31 %	19	12 %	11	7 %
**Total**						1477	20.0 %	540	7.3 %	234	3.2 %

The 10-year risk of breast cancer was calculated using the CanRisk tool (https://www.canrisk.org/), considering women aged 40 and varying different risk factors.

A body mass index (BMI) of 23 was obtained with a height of 162 cm and a weight of 62 kg, a BMI of 30 was obtained with a height of 176 cm and a weight of 93 kg.

The duration of use of contraceptive pill was set at 10 years.

The age of first pregnancy has been set at 34 for women with children.

Breast density was encoded according to BI-RADS categories.

According to the British National Institute for Health and Care Excellence, 10-year breast cancer risk was considered near-population when it was less than 3 %, moderate when it was between 3 % and 8 %, and high when it was greater than 8 % [18].

## 4 Discussion

The introduction of PRS into clinical practice is debated, and studies are still needed on the effectiveness of personalized screening programs. However, there is little doubt that PRS will one day be required in clinical practice for personalized breast cancer risk calculation. Our optimized panel makes it possible to calculate a polygenic risk score by targeted NGS, with very good correlation with the reference score calculated by SNP array (R^2^ coefficient >0.95). This method has sensitivity and specificity of over 95 %. Above all, we were able to show that our method of determining PRS by NGS only alters the breast cancer risk category for at most 3 % of our patients, whose PRS is close to the thresholds.

The possibility of calculating PRS using NGS means that a single technique could be used to calculate PRS and sequence panels of predisposition genes. This would save oncogenetic laboratories time, money and the investment and expertise required for SNP arrays. The NGS technique is less flexible, since unlike SNP array, it is not possible to recalculate the PRS in the event of a change in the relevant PRS. Nevertheless, as diagnostic gene panels are also rapidly evolving, most oncogenetics laboratories regularly update their diagnostic panel. In this case, NGS panel PRSs can be reviewed regularly to incorporate the latest published PRSs, including any ethnicity-dependent PRSs.

Most of the genotype differences observed between array PRS and NGS PRS are on imputed SNPs. When the SNPs are not located in a low-complexity region, our data show sufficient quality to assume that the sequencing is correct, and that the imputation should be challenged. In such cases, the genotype defined by NGS is probably more accurate than the imputed genotype, as already shown [19]. Our NGS method enables to directly genotype 289 of the 313 SNPs, whereas conventional microarrays require imputation of a majority of SNPs. However, the predictive value of PRS in breast cancer have been demonstrated on very large cohorts using iCOGS and OncoArray chips [1,2]. Even if some of the imputed SNPs could be more accurate in NGS, the array PRS thus remains the standard reference. However, it would be interesting to test on a large cohort whether our NGS PRS with more accurate genotypes would improve the prediction of breast cancer risk compared with the OncoArray PRS_313_, which includes a large proportion of imputed SNPs.

Another limitation of the NGS technique concerns regions of low complexity (homopolymers, repeated regions), which are always difficult to sequence. To this end, we designed 27 new SNPs, in linkage disequilibrium with poorly sequenced SNPs, which were added to our NGS panel. We were able to show that this optimized PRS panel considerably improves the correlation of our NGS PRS with the PRS obtained with the OncoArray.

Other PRS have already been designed for NGS: the BRIDGES PRS is an adaptation of PRS_313_ for NGS that consists of 295 designed SNPs and 11 SNPs in linkage disequilibrium (r^2^ > 0.9 in Europeans) [11]. 18 SNP from PRS_313_ are missing from the BRIDGES 306 PRS. The PERSPECTIVE I&I PRS was designed in Ontario and Quebec as an NGS panel: 287 of 313 markers could be designed and a further 8 were surrogates [20]. Baumann et al. described an NGS-based PRS genotyping in German centers [12]. They compared the allelic frequencies of SNPs obtained from at least 100 women in each center with the expected allelic frequencies in the general gnomAD population. For 11 SNPs of the PRS_313_, allelic frequencies deviated significantly, and they suggest using alternative variant callers or proxy variants for these loci. However, this study involved people with familial breast and ovarian cancers, which constitutes a significant bias since allelic frequency deviations could be due to genetic load rather than technical artifacts.

On the contrary, our study is the only one, to our knowledge, to have directly compared a PRS_313_ panel optimized for NGS with PRS_313_ genotyped by OncoArray, on over 150 patients. We have been able to validate that our optimized PRS panel can calculate the PRS_313_ with an analytical sensitivity and specificity of over 95 %. In conclusion, this panel is therefore ideally suited to facilitating routine implementation of the PRS_313_ in clinical oncogenetics laboratories. Spiking this design into an existing routine HBOC panel is a time- and cost-effective way to provide PRS to patients as it would add only minimal sequencing increase and no benchwork.

## CRediT authorship contribution statement

**Flora Ponelle-Chachuat:** Writing – review & editing, Writing – original draft, Visualization, Validation, Software, Resources, Project administration, Methodology, Investigation, Formal analysis, Data curation, Conceptualization. **Mathis Lepage:** Writing – review & editing, Visualization, Validation, Supervision, Project administration, Methodology, Investigation, Data curation, Conceptualization. **Sandrine Viala:** Writing – review & editing, Visualization, Validation, Methodology, Formal analysis, Data curation, Conceptualization. **Mikaïl Kelleci:** Software, Methodology, Formal analysis, Data curation. **Edith Le Floch:** Writing – review & editing, Visualization, Validation, Methodology, Formal analysis, Data curation. **Claire Dandine-Roulland:** Writing – review & editing, Visualization, Validation, Methodology, Formal analysis, Data curation. **Delphine Bacq:** Writing – review & editing, Visualization, Validation, Methodology, Formal analysis, Data curation. **Robert Olaso:** Writing – review & editing, Visualization, Validation, Methodology, Formal analysis, Data curation. **Jean-François Deleuze:** Writing – review & editing, Visualization, Validation, Project administration, Methodology, Formal analysis, Data curation. **Nancy Uhrhammer:** Writing – review & editing, Visualization, Validation, Resources, Methodology, Formal analysis, Data curation, Conceptualization. **Mathilde Gay-Bellile:** Writing – review & editing, Validation, Supervision, Project administration, Methodology, Investigation, Formal analysis, Conceptualization. **Yannick Bidet:** Writing – review & editing, Writing – original draft, Visualization, Validation, Supervision, Software, Resources, Project administration, Methodology, Investigation, Funding acquisition, Formal analysis, Data curation, Conceptualization. **Maud Privat:** Writing – review & editing, Writing – original draft, Visualization, Validation, Supervision, Software, Resources, Project administration, Methodology, Investigation, Funding acquisition, Formal analysis, Data curation, Conceptualization.

## Ethical approval

All patients signed informed consent, and study ethics approval was obtained on June 24, 2024 (IRB committee of CJP, IRB 00013468).

## Declaration of competing interest

The authors declare that they have no known competing financial interests or personal relationships that could have appeared to influence the work reported in this paper.

## Figures and Tables

**figs1:**
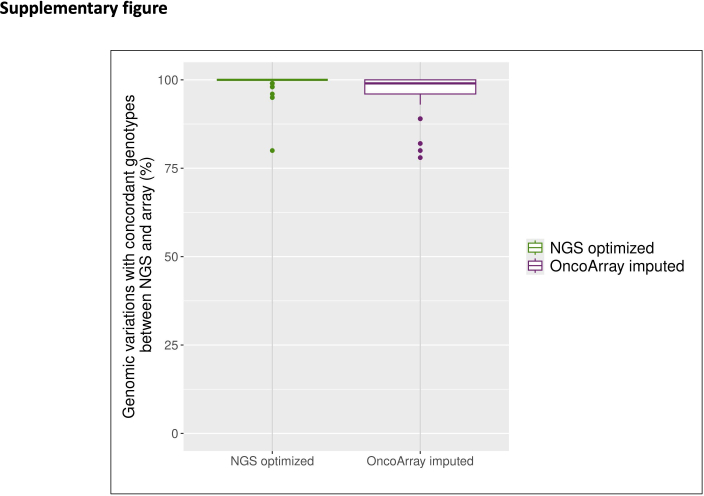

